# INFECTIOUS DISEASE CONTROL REGULATIONS IN ASSISTED LIVING COMMUNITIES: BEFORE AND AFTER COVID-19

**DOI:** 10.1093/geroni/igae098.3858

**Published:** 2024-12-31

**Authors:** Danielle Gerken, Lindsey Smith, Paula Carder, Cassandra Hua, Eric Jutkowitz, Kali Thomas

**Affiliations:** Oregon Health & Science University, Portland, Oregon, United States; Oregon Health & Science University, Portland, Oregon, United States; OHSU-PSU School of Public Health, Portland, Oregon, United States; University of Massachusetts Lowell, Lowell, Massachusetts, United States; Brown University, Brown University, Rhode Island, United States; Johns Hopkins University, Baltimore, Maryland, United States

## Abstract

Assisted living (AL) residents are at high risk for adverse health outcomes due to infection, particularly those living in memory care. Despite this, at the outset of the COVID-19 pandemic, most states had minimal or no regulations regarding infectious disease control within AL communities. In this study, we sought to determine if state agencies responded to the COVID-19 pandemic by increasing infectious disease control regulations for AL. We systematically sourced AL regulations for all 50 states and Washington D.C. from the legal policy database Westlaw. We then used search-term driven text retrieval to identify sections of policy relevant to infection control to determine the applicability and scope of each policy. In 2019, there were 13 regulations from 9 states regarding how AL providers should respond to pandemics and epidemics. In 2020, 13 regulations from 10 different states were found. In 2023, this number increased to 18 regulations from 11 different states. The majority of regulations required AL providers to inform a local health department or official about any disease outbreak occurring within the facility. In 2019, 7 of the 9 states (77.8%) had regulations requiring reporting. In 2020, that number decreased to 6 out of the 10 states (60%), and in 2023 the number of states requiring reporting was 8 out of the 11 (72.7%). Our analysis revealed that while a few states have made improvements, there is still a gap in regulatory protection that could result in poor health outcomes for AL residents.

